# Effectiveness and compliance to the use of sulphadoxine-pyrimethamine as a prophylaxis for malaria among pregnant women in Port Harcourt, Rivers State, Nigeria

**DOI:** 10.4314/ahs.v22i2.22

**Published:** 2022-06

**Authors:** Helen Onoja, Florence O Nduka, Austin E Abah

**Affiliations:** Department of Animal and Environmental Biology, Faculty of Science, University of Port Harcourt. Rivers State, Nigeria

**Keywords:** Compliance, Sulphadoxine-Pyrimethamine, Pregnant women, Malaria Parasitaemia

## Abstract

**Background:**

Malaria during pregnancy escalates the damaging consequence to the mother and neonate. The usage of intermittent preventive treatment of malaria (IPTp) with sulfadoxine-pyrimethamine (SP) is recommended for averting the deleterious consequences of malaria in pregnancy. This study evaluated the effectiveness of, and compliance with the use of SP for malaria among pregnant women in Port Harcourt Rivers State, Nigeria.

**Method:**

A total of 300 samples of maternal peripheral blood (MPB), 84 neonatal cord blood (NCB) and 84 placental blood (PLB) were collected from consenting mothers. Malaria parasitaemia were analysed using standard parasitological methods, and bio-data of consenting mothers were collected through questionnaires and from ANC records.

**Results:**

Out of the samples examined for MPB, 59(19.7%) tested positive to malaria. Those with only primary education (57.1%) and women of age ≤ 20yrs (25%) had higher prevalence. Women who took SP had significantly lower prevalence (17.6%) than those that took other drugs (36.4%) (p < 0.05). Malaria prevalence was highest among women who had 3 months interval between each dose (39.1%), followed by those of 2months (23.7%) and those of 1 month (7.0%) (p < 0.05). The primigravidaes (22.8%) had an insignificantly higher prevalence than secundigravidae (19.4%) and multigravidae (15.9%). Also, 30.5% of women who registered in their third trimester of pregnancy had a significantly higher malaria parasitaemia than those who registered during their first 8.10%, or second trimesters, 19.4%. Of the 84 MPB-NCB-PLB pairedamples examined, 16.7%, 8.3% and 25% respectively were infected with malaria parasitaemia. On frequency of compliance, mothers who took SP once (37.5%) had a significantly higher MPB parasitaemia than those who took it twice (7.84%) and those of thrice (6.25%). Neonatal cord blood parasitaemia prevalence revealed that those that took SP once, that is, 25%, had a higher prevalence than others like those of twice (5.88%) and thrice (0%) respectively.

**Conclusion:**

The use and compliance of SP reduced the prevalence of malaria among pregnant women and their new-borns.

## Introduction

About 32 million pregnancies occur annually in Africa's malarious regions^1^, and 74 % of this population live in areas that are highly endemic for malaria^2^. Malaria during pregnancy is of great public health importance much as it is associated with serious adverse consequences such as maternal deaths, abortion, premature labour, maternal anaemia and low birth weight resulting in maternal and fetal morbidity and mortality. Coupled with the foregoing is that annual average of 200,000 infant deaths has also been associated with malaria in pregnancy^3^. These unfavorable pregnancy outcomes are connected with sequestration of malaria parasites in the placental intervillous spaces attached to chondroitin-sulphate-A^4^,^5^. When Pro-inflammatory cells and cytokines also invade the placental bed, the net result is impairment of foetal blood and nutrient supply, which in turn results in low birth weight (LBW), and most times this proves the greatest risk factor for neonatal mortality as a major contributor to infant mortality^5^. The pregnant woman runs a higher risk of contracting malaria than her non-pregnant counterpart^6^,^7^. A short/occasional drop or reduction in the maternal immunity due to pregnancy is one of the reasons adduced for the increased susceptibility of the pregnant woman to malaria. Although malaria in pregnancy is often asymptomatic, it is however the cause of a number of unfavorable pregnancy outcomes both in the mother and in her baby^6^,^8^. The African Summit on Roll Back Malaria in April 2000 adopted the Abuja Declaration in which regional leaders committed themselves to ensuring that 60 % of pregnant women in malaria-endemic communities accessed effective prevention and treatment of malaria by 2005^9^. Also 80% scale-up was also initiated by the federal government to ensure that at least 80% of pregnant women in the country participate in IPTp-SP^10^.

Over the years, malaria prophylaxis with pyrimethamine or chloroquine was broadly adopted in many African countries^11^. However, poor compliance and emergence of drug resistant strains of Plasmodium falciparum compromised the efficacy of these drugs^12^. In order to reduce the possibility of Plasmodium falciparum infection in pregnancy and its subsequent adverse effects, the World Health Organization (WHO) recommends intermittent preventive treatment in pregnancy using sulphadoxine-pyrimethamine (IPTp-SP) with at least 2 doses of SP one month at intervals after quickening^13^. IPTp-SP is provided as part of a comprehensive antenatal package to control maternal anaemia, and it has proved to be safe, inexpensive and effective^14^; and it has resulted in increase of both maternal haemoglobin levels and the infant's birth weight^15^,^16^. Although majority of the pregnant women attend antenatal clinic at least once during pregnancy, extant indication shows that IPTp-SP uptake as well as ITN coverage among pregnant women is unacceptably low in most countries^17^, and lowest in areas with highest transmission of malaria^18^

While most pregnant women in Nigeria receive IPTp-SP, at least once during pregnancy about 5% of them take it up to three times^19^. This study is intended to aid relevant programmes and policies needed to ensure a universal and an optimal coverage of IPTp-SP. The study ultimately aimed at evaluating the effectiveness of, the use of SP as a preventive treatment in a Tertiary Hospital in Port Harcourt.

## Methods

### Study Area

This was a cross-sectional descriptive study carried out between February and June, 2018 among pregnant women who registered for antenatal at Rivers State University Teaching Hospital (RSUTH), and those who had their babies in their labour ward. Port Harcourt is the capital of Rivers State which is one of the largest cities in the Niger Delta region of Nigeria. The temperature all through the year varies between 25°C to 32°C.

### Inclusion and Exclusion Criteria

Healthy Women who attended ANC in RSUTH Labour ward and who consented to be studied with their Neonates enrolled for the study. This study excluded mothers who are suffering from Sickle Cells Disease (SCD), Human Immunodeficiency Virus (HIV) and Diabetics. Healthy mothers who refused to consent to be studied were excluded from the samples, and as such were completely excluded in the overall research study.

### Ethical Clearance

Permission was obtained from Rivers State Ministry of Health, Rivers State Hospital Management Board, the Chief Medical Director of the Rivers State Teaching Hospital and University of Port Harcourt Ethical Committee. Each mother's consent was also obtained.

### Administration of Questionnaire

A designed proforma containing obstetrics and demographic questions relating to age, education, parity, types of preventive drug taken during the pregnancy and other personal efforts to prevent malaria was administered to the women. This study proforma was quickly done during the first stage of labour. Other useful information was gotten from their ANC records.

### Sample Collections

Maternal Peripheral Blood (MPB) samples were collected from 300 consenting mothers, while only 84 women consented to the collection of Neonatal Cord Blood (NCB) and Placental Blood (PLB). All samples were collected into Ethylene Diamine Tetra Acetic Acid (EDTA) bottles to prevent clotting. Samples were then transported to the Parasitology Research Laboratory of the Department of Animal and Environmental Biology for analysis. The mothers' 5mls pre-delivery peripheral blood was taken on admission into the labour ward. The cord blood of the neonates and Placental blood of consenting mothers were collected immediately after the delivery into heparinized tubes respectively.

### Laboratory Analysis

Thick and thin blood films for each sample were prepared on a clean grease-free microscope glass slide. The smears were air dried. The thin films were fixed in absolute methanol and they were both stained in 1:10 dilution of Giemsa stain. They were air-dried and examined under x100 objective lens of the binocular light Microscope^20^.

### Data Analysis

Data obtained from the study were presented for analysis using SPSS version 22. Chi-square trend (χ^2^) with Yate's correction was used to investigate the effects of quantitative and qualitative variables respectively. A p-value <0.05 was considered significant.

## Results

Of the 300 women examined, 59(19.66%) tested positive to malaria with those that had primary education (57.1%) having a significantly higher prevalence of infection than those of secondary (29%) and those of tertiary (16.5%) counterparts (χ^2^=8.101, df=2, p=0.017) as shown in Table 1.

**Table 1:** Relationship between plasmodium parasitaemia and social dermographic data among pregnant women in Port Harcourt

Characteristic	No. Examined	No Infected	χ^2^	p-value
**Educational Status**				
Primary	7	4(57.1)		
Secondary	105	24(22.9)		
Tertiary	188	31(16.5)	8.101	0.017
Total	300	59(19.66)		
**Ages (Yrs)**				
≤ 20	12	3(25)		
21–30	109	23(21.10)		
31–40	161	30(18.63)		
≥ 41	18	3(16.66)	0.569	0.903
**Parity**				
Primigravidae	114	26(22.80)		
Secundigrvidae	98	19(19.38)		
Multigravidae	88	14(15.9)	1.503	0.472

Those aged ≤20 yrs (25%) had the highest prevalence followed by those of 21–30yrs (21.10%) while those of >40yrs (16.66%) had the least. And although there was difference in prevalence across the various age groups, it was not significant (χ^2^=0.569, df=3, p=0.903).

Parity of the participants showed that the primigravidaes (22.80%) had an insignificantly higher prevalence than secundigravidae (19.38%) and multigravidae (15.9%) (χ^2^=2.728, df=2, p=0.472) (Table 1). Women (30.49%) who registered in their third trimester of pregnancy had a significantly higher malaria parasitaemia than those who registered during either their first 8.10%, or second trimesters, 19.44%, (χ^2^=12.340, df=2, p=0.002). Preventive drugs taken during the pregnancies showed that 17.60% of women who took SP had a significantly lower prevalence compared to women who took other drugs, i.e.36.36% (χ^2^=6.543, df=1, p=0.011) (Table 2). The trimester in which the usage of SP started revealed that 14.86% of the pregnant women who started treatment in their second trimesters had an insignificantly lower infectionhan 25% who started in their third trimester (χ^2^=3.719, df=1, p=0.054).

**Table 2:** Relationship between parasitaemia and IPTP-SP compliance among pregnant women in Port Harcourt

Time of ANC registration	No. Examined	No Infected	χ^2^	p-value
First	74(24.7)	6(8.10)		
Second	144(48)	28(19.44)		
Third	82(27.3)	25(30.49)	12.340	0.002
**Preventive Drugs**				
SP	267(89)	47(17.60)		
Others	33(11)	12(36.36)	6.543	0.011
**Trimester SP** **intake started**				
Second	195 (73)	29(14.86)		
Third	72 (27)	18(25)	3.719	0.054
**Number of times** **SP was taken**				
Once	40(14.9)	11(27.5)		
Twice	204(76.4)	34(16.7)		
Thrice	23(8.6)	2(8.69)	4.083	0.130
**Interval between** **intake of doses of** **SP**				
1 month apart	128 (56.39)	9(7.03)		
2 months apart	76 (33.48)	18(23.68)		
3 months apart	23 (10.1)	9(39.13)	20.297	0.000

Malaria parasitaemia, in relation to the number of times SP was taken, indicated that pregnant women who took SP once had a prevalence of 27.5%, while those that took it twice and thrice had prevalence of 16.7% and 8.69% respectively. In this connection, the difference was insignificant (χ^2^=3.897, df= 2, p=0.143).

Malaria infection with respect to the interval between intake of SP revealed that prevalence was highest among those who had 3 months interval between each dose (39.13%), followed by those of 2months (23.68%); while those that took at 1 month interval had the lowest (7.0%), and the difference between them was significant (χ^2^=13.047, df=2, p= 0.000) (Table 2).

Eighty-four (84) women consented to the collection of their three blood samples (MPB, NCB and PLB) out of which 75 were on SP and 9 were not. The result revealed that 33.33% of those not on SP had an insignificantly higher prevalence of maternal peripheral blood (MPB) parasitaemia than those on SP which constituted (14.66) (χ^2^=2.016, df=1, p=0.156). Neonatal cord blood examination also showed that those not on SP, the 22.22% had a higher prevalence of infection than those on SP (6.66%), but the difference was insignificant (χ^2^=2.545, df=1, p=0.111). Malaria prevalence in the placental blood revealed that those not on SP made up of66.66% had a significantly higher prevalence than their SP counterparts totalling 20%, (χ^2^=9.333, df=, p=0.002). (Table 3)

**Table 3:** Malaria parasitaemia based on compliance to sp among pregnant women in Port Harcourt

SP compliance	No. Examined	No. Infected		
		MPB(%)	NCB(%)	PLB(%)
Compliant	75	11(14.66)	5(6.66)	15(20)
Non-compliant	9	3(33.33)	2(22.22)	6(66.66)
Total	84	14(16.66)	7(8.33)	21(25)
χ^2^		2.016	2.545	9.333
p-value		0.156	0.111	0.002
**SP-compliants usage** **frequency**				
Once	8	3(37.5)	2(25)	4(50)
Twice	51	4(7.84)	3(5.88)	10(19.61)
Three	16	1(6.25)	0(0)	1(6.25)
Total	75	8(10.66)	5(6.66)	15(20)
χ^2^		6.799	5.515	6.396
p-value		**0.033**	**0.063**	**0.041**

* Only 84 participants gave consents for the collection of the three samples (MPB-Maternal Peripheral Blood, NCB-Neonatal Cord Blood and PLB-Placenta Blood.

Based on frequency of compliance, mothers who took SP once (37.5%) had a significantly higher MPB parasitaemia than those who took it twice (7.84%) and those of thrice (6.25%) (χ^2^=6.799, df=2, p=0.033). Neonatal cord blood parasitaemia prevalence revealed that those that took SP once, had a higher prevalence (25%) than those that took twice (5.88%) and thrice (0%) respectively; but the difference was insignificant χ^2^=5.515, df=2, p=0.063, while parasitaemi prevalence in PLB showed a significant difference between those whose frequencies were once 50%, twice 19.61% and thrice 6.25% (χ^2^=6.396, df=2, p=0.041) as shown in Table 3

## Discussion

The results of this study indicated that SP use during pregnancy is effective in reducing malaria prevalence both in the maternal peripherial blood (MPB) and Neonatal cord bloood as mothers who took SP onlyonce (37.5%) had a significantly higher MPB parasitaemia than those who took it twice (7.84%) and those of thrice (6.25%). Also, neonatal cord blood parasitaemia prevalence revealed that those that took SP once, had a higher prevalence (25%) than those that took twice (5.88%) and thrice (0%) respectively. This finding agrees with the findings in Kenya and Nigeria^17^,^21^.

In an evaluation of the effectiveness of SP in preventing maternal malaria, it was observed that women who took SP had a significantly lower prevalence compared to women who took other drugs. A prevalence of 17.6% was recorded in this study among SP users which agrees with a prevalence of less than 20% reported^22^. This shows the protective effects of SP, as the non SP compliant had a significantly higher prevalence value.

The use of SP as an approved preventive drug was high since 89% of the women were Sp-complaints. This value agrees with the findings of Bassey who reported 85.7% in the same region^23^. This value however varies with the 33% reported in the eastern part of Nigeria^21^. The higher compliant level in this study area could be attributable to a number of factors such as the availability of the drug, the type of health facility, the social economy class of the women in the study area, the health information and education of the women on malaria prevention in pregnancy. These results also indicated that the national 80% scale-up target of IPTp-SP in the study area had been met. The 89% recorded in the current study could be as a result of progress made by the federal government in developing policy thrust, partnership and funding necessary for the effective malaria control in pregnancy. The recommended doses of IPTp in Nigeria are three doses. However, less than half took the complete three doses, probably due to late registration for antenatal care at the third trimester (48%), skipping of ANC appointment and because they do not enforce the Directly Observed Therapy (DOT), or due to failure of some of the women to take their drugs.

Approximately, 15% of the women took SP once during pregnancy while 76.4% took it twice and 9% took it thrice. These findings are similar to the report of Doku and Mpungu who discovered that less than half pregnant women took the recommended doses in Ghana and Uganda respectively^24^,^25^. The interval between successive doses of SP indicated that 63% of the women took the doses one month interval. This relatively high level of adherence could be attributed to the numerous efforts made by the government and Hospital management to ensuring compliance to WHO recommendations pertaining SP usage. The significant decrease in maternal parasitaemia between those that took their doses one month apart and others could be due to the possibility of higher concentration of SP circulating in their peripheral blood. The non-total compliant attitude of women towards taking complete SP dosages may be the reason why tis study recorded 19.7% malaria parasitaemia despite that we recorded 89% compliance to SP.

Out of the 300 women that were recruited into this study, only 84 women gave their consent for the collection of their NCB and PLB. Of the 84 MPB-NCB-PLB paired samples examined, 16.66%, 8.33% and 25% respectively were infected with malaria parasitaemia. There was no significant associations of parasitaemia between SP-compliants (14.66%) and non-compliants (33.33%) in MPB (p>0.05), and SP-compliants (6.66%) and non-compliants (22.22%) in NCB (p>0.05) but a significant association existed between them in PLB (p<0.05). The result showed that it was not in all cases of Placental Parasitaemia that Neonatal Parasitaemia was discovered and this resulted to a lower prevalence of 22% and 6.7% for those on SP and those not on SP respectively. This may be because of the Foetus IgG antibodies which help to fight against diseases and only densely positive cases of Placental parasitaemia can transfer to the foetus^23^.

The women who used SP as preventive drug in pregnancy recorded a significantly lower malaria prevalence than those that took other drugs. Likewise women that complied strictly with the rules of SP recorded 0% parasitaemia in the Neonatal Cord Blood films. This again buttresses the fact that SP is very effective in malaria prevention.

## Comclusion

SP-compliant women recorded significantly lower maternal and Neonatal malaria infections and also lower malaria-related adverse pregnancy effects. Intermittent preventive treatment in pregnancy using Sulphadoxine-pyrimethamine (IPTp-SP) is an effective agent against malaria in pregnancy. There is also increased response to the use of SP as an ant-malarial agent in pregnancy. This means that the federal government campaign and funding of the programme is yielding positive results in the study area. However to ensure total compliance, the campaign on early registration for ANC should be intensified as some women deliberately choose to register late. The health education on the importance of not postponing ANC appointment should be intensified and the Pharmacist or Midwife should ensure that the Directly Observed Therapy rule is adhered to strictly.
